# Differences between Frequentist and Bayesian inference in routine surveillance for influenza vaccine effectiveness: a test-negative case-control study

**DOI:** 10.1186/s12889-021-10543-z

**Published:** 2021-03-16

**Authors:** Michael L. Jackson, Jill Ferdinands, Mary Patricia Nowalk, Richard K. Zimmerman, Burney Kieke, Manjusha Gaglani, Kempapura Murthy, Joshua G. Petrie, Emily T. Martin, Jessie R. Chung, Brendan Flannery, Lisa A. Jackson

**Affiliations:** 1 Kaiser Permanente Washington Health Research Institute, 1730 Minor Ave, Suite 1600, Seattle, WA 98101-1448 USA; 2 Centers for Disease Control and Prevention, Atlanta, GA USA; 3 University of Pittsburgh School of Medicine, Pittsburgh, PA USA; 4 Marshfield Clinic Research Institute, Marshfield, WI USA; 5 Baylor Scott & White Health, Temple, TX USA; 6 Texas A&M College of Medicine, Temple, TX USA; 7 University of Michigan School of Public Health, Ann Arbor, MI USA

**Keywords:** Influenza, Influenza vaccine, Effectiveness, Test-negative case-control design, Bayesian statistics, Frequentist statistics

## Abstract

**Background:**

Routine influenza vaccine effectiveness (VE) surveillance networks use frequentist methods to estimate VE. With data from more than a decade of VE surveillance from diverse global populations now available, using Bayesian methods to explicitly account for this knowledge may be beneficial. This study explores differences between Bayesian vs. frequentist inference in multiple seasons with varying VE.

**Methods:**

We used data from the United States Influenza Vaccine Effectiveness (US Flu VE) Network. Ambulatory care patients with acute respiratory illness were enrolled during seasons of varying observed VE based on traditional frequentist methods. We estimated VE against A(H1N1)pdm in 2015/16, dominated by A(H1N1)pdm; against A(H3N2) in 2017/18, dominated by A(H3N2); and compared VE for live attenuated influenza vaccine (LAIV) vs. inactivated influenza vaccine (IIV) among children aged 2–17 years in 2013/14, also dominated by A(H1N1)pdm. VE was estimated using both frequentist and Bayesian methods using the test-negative design. For the Bayesian estimates, prior VE distributions were based on data from all published test-negative studies of the same influenza type/subtype available prior to the season of interest.

**Results:**

Across the three seasons, 16,342 subjects were included in the analyses. For 2015/16, frequentist and Bayesian VE estimates were essentially identical (41% each). For 2017/18, frequentist and Bayesian estimates of VE against A(H3N2) viruses were also nearly identical (26% vs. 23%, respectively), even though the presence of apparent antigenic match could potentially have pulled Bayesian estimates upward. Precision of estimates was similar between methods in both seasons. Frequentist and Bayesian estimates diverged for children in 2013/14. Under the frequentist approach, LAIV effectiveness was 62 percentage points lower than IIV, while LAIV was only 27 percentage points lower than IIV under the Bayesian approach.

**Conclusion:**

Bayesian estimates of influenza VE can differ from frequentist estimates to a clinically meaningful degree when VE diverges substantially from previous seasons.

**Supplementary Information:**

The online version contains supplementary material available at 10.1186/s12889-021-10543-z.

## Background

Globally, a number of surveillance networks provide annual estimates of influenza vaccine effectiveness (VE) against laboratory-confirmed influenza disease diagnosed in ambulatory or inpatient settings [1–5]. These networks aim to estimate VE both overall and stratified by various factors including age, virus subtype/lineage, and vaccine type. VE is estimated through frequentist statistical methods, wherein event probabilities are treated as expected frequencies were the study to be repeated many times in some hypothetical population. One hallmark of frequentist methods is the desire to avoid subjectivity on the part of the researcher, such that information from outside the study is not considered when estimating parameters and assigning uncertainty to the estimates [6].

The frequentist paradigm has proven its utility in many settings and has the benefit of familiarity to diverse audiences. However, the influenza research community now collectively has more than a decade’s worth of influenza VE estimates drawn from diverse populations worldwide. These studies inform our expectations about influenza VE before annual estimates are computed. The Bayesian statistical paradigm, in which probabilities are considered to be beliefs about the likelihood of an outcome, provides a framework by which information from prior VE studies can be explicitly incorporated into VE estimates for the current season [7]. Incorporating data from previous studies may enable us to more precisely estimate VE with smaller sample sizes, which would be useful both for providing early-season VE estimates and for estimating VE among sub-groups.

However, a potential challenge to the Bayesian approach is that VE can vary in ways that are not necessarily predictable a priori due to unexpected issues with vaccine potency, antigenic match, or other factors such as vaccine coverage and delay/shortage. Defining Bayesian priors from previous studies could potentially lead to incorrect inference in these settings, as these priors could potentially lead to posterior estimates of VE that suggest effectiveness even in mismatch years. To assess this possibility, we compare how the use of Bayesian vs. frequentist methods may affect the inferences we would draw about influenza VE from surveillance networks.

## Methods

### Study setting

This study was conducted using data from the United States Influenza Vaccine Effectiveness (US Flu VE) Network, the details of which have been described previously [1, 8]. Briefly, US Flu VE Network institutions enroll patients with acute respiratory illness (cough of < 8 days’ duration) from ambulatory care sites. Subjects provide paired nasal and oropharyngeal swab specimens (nasal swabs only in subjects aged < 2 years), which are tested for influenza via real-time reverse transcriptase polymerase chain reaction (RT-PCR).

The current iteration of the US Flu VE Network has been in operation since the 2011/12 influenza season. In this study, we estimated influenza VE during three specific seasons, chosen to capture some key aspects of heterogeneity in influenza VE, based on antigenic match between vaccine and circulating virus strains and estimated VE from frequentist methods:

- Effectiveness against A(H1N1)pdm viruses during the 2015/16 influenza season, which was dominated by A(H1N1)pdm and for which overall VE (across age groups) was consistent with expectations, given antigenic similarity between circulating and vaccine viruses [1, 9];
- Effectiveness against A(H3N2) viruses during the 2017/18 influenza season, which was dominated by A(H3N2) and for which overall VE was lower than expected, given antigenic similarity between circulating and vaccine viruses [10, 11];
- Effectiveness of live attenuated influenza vaccine (LAIV) against A(H1N1)pdm in children aged 2–17 years during the 2013/14 season, which was unexpectedly lower than effectiveness of inactivated influenza vaccines (IIV) that season [12].

### Exposure and outcome

Study staff collected subject information, including influenza vaccination history, through interviews at enrollment and extraction from healthcare databases and other electronic data sources. The exposure of interest was receipt of seasonal influenza vaccine at least 14 days prior to illness onset. Vaccine receipt was defined by electronic immunization records, which included electronic health records, employee health records, and state immunization registries. Subjects vaccinated < 14 days prior to illness onset were excluded from the study.

The outcome of interest was laboratory-confirmed influenza infection. Subjects with swabs testing positive for influenza by RT-PCR were classified as cases, while subjects testing negative were classified as non-cases. Subjects with inconclusive RT-PCR results were excluded. To avoid bias due to sampling non-cases outside of influenza season [13], we excluded non-cases who were enrolled prior to the first or after the last detected influenza case at each site in the 2013/14, 2015/16, or 2017/18 influenza seasons. We excluded subjects with laboratory-confirmed influenza A(H3N2) or B infection in 2013/14 and 2015/16 or with laboratory-confirmed A(H1N1)pdm or B infection in 2017/18. Note that these criteria are somewhat simplified from the exclusion criteria in the original studies.

### Statistical analysis

Within each study year, we compared the distribution of influenza vaccination and of study covariates between cases and non-cases, using proportions for categorical variables and means/standard deviations for continuous variables.

We used a test-negative design to create frequentist estimates of influenza VE (VE_F_). In this design VE_F_ was estimated as (1-OR_F_), where OR_F_ is the vaccination odds ratio for cases vs. non-cases from a logistic regresion model, with parameters estimated using maximum likelihood [14]. Consistent with prior US Flu VE Network studies, models were adjusted for study site, age and date of illness onset (both using linear tail-restricted cubic splines), and presence of high-risk medical conditions. Separate estimates were produced for each season. We computed 95% confidence intervals (CIs) for these estimates.

To create Bayesian estimates of influenza VE (VE_B_) we first defined prior distributions for all model parameters. Prior distributions for baseline prevalence of any influenza infection (i.e. the model intercept) and for associations between covariates and case/non-case status were generated from all previous seasons’ data from the US Flu VE Network. For example, prior distributions for the association between age group and influenza odds in the 2015/16 season were generated from the age/influenza odds during the 2011/12 through 2014/15 seasons.

To create the prior distribution for the vaccination odds ratio, we first reviewed the literature to identify published estimates of influenza VE. We restricted the previous studies to peer-reviewed publications using RT-PCR-confirmed endpoints, and only end-of-season, fully adjusted VE estimates were included (Supplemental Appendix). For each of the three scenarios of interest, we selected all VE estimates from seasons before the relevant influenza season from subjects of the same age range and against the same virus sub-type. For example, the prior distribution of VE against A(H3N2) in 2017/18 was informed by estimates of VE against A(H3N2) among persons of any age (≥6 months) from Northern Hemisphere 2016/17 or Southern Hemisphere 2017 and earlier. These published VE estimates and associated standard errors were converted to Normally distributed regression coefficients [as log (1-VE)]. Finally, we created prior distributions by stochastically sampling values from these coefficient distributions, weighted by study sample size. We did not attempt to create separate priors for IIV vs. LAIV for the 2013/14 LAIV vs. IIV comparions, since as of 2013/14 there was little reason to expect lower VE for LAIV.

After creating the priors, VE_B_ was estimated as 1-OR_B_, where OR_B_ is the posterior odds ratio from a generalized linear model using a binomial distribution and logistic link, adjusted for the same covariates as the frequentist estimates. Posterior values were estimated using Gibbs sampling with 1000 burn-in iterations and 10,000 sampling iterations [15, 16]. We assessed convergence of the Markov chains by confirming stationarity of trace plots and lack of auto-correlation between sampled values. We computed 95% Bayesian credible intervals (BCIs) for these estimates. We also estimated parameters assuming a noninformative (i.e. uniform) prior.

In addition to estimating VE_F_ and VE_B_ using end-of-season data, we assessed the impact of sample size on the precision of VE estimates. For this analysis, we estimated VE in the 2015/16 and 2017/18 seasons at progressively increasing enrollment sizes, starting with the first 25 cases enrolled, then the first 50 cases enrolled, and proceding in increments of 25 until the full sample size was reached. Similarly, we estimated VE based on total enrollment using increments of 100. Analyses were performed using SAS version 9.4 (SAS Institute Inc., Cary NC) and R version 3.6.1 (The R Foundation for Statistical Computing, Vienna Austria).

## Results

The US Flu VE Network had 18,084 enrollees for the relevant seasons and age groups (1621 children aged 2–17 years in 2013/14; 7563 enrollees of all ages in 2015/16; 8900 enrollees of all ages in 2017/18). Of these, 2290 were excluded (87 for inconclusive RT-PCR results; 174 non-cases enrolled before the first or after the last case; 126 vaccinated < 14 days before illness onset; 44 infected with A(H3N2) or B in 2013/14, 552 infected with A(H3N2) or B in 2015/16, and 1307 infected with A(H1N1)pdm or B in 2017/18), leaving 15,794 subjects for analysis (Table 1).

**Table 1:** Characteristics of eligible United States influenza vaccine effectiveness enrollees in each study year

Variable	Category	2013/14 A(H1N1)pdm	2015/16 any influenza	2017/18 A(H3N2)
Total		1542	7372	7428
Site	MI	245 (16%)	1076 (15%)	1257 (17%)
	PA	262 (17%)	1820 (25%)	1188 (16%)
	TX	297 (19%)	1395 (19%)	1738 (23%)
	WA	256 (17%)	1825 (25%)	1418 (19%)
	WI	482 (31%)	1256 (17%)	1827 (25%)
Age group	< 5 years	512 (33%)	1148 (16%)	1193 (16%)
	5–8 years	433 (28%)	631 (9%)	560 (8%)
	9–17 years	597 (39%)	898 (12%)	920 (12%)
	18–49 years	excl^a^	2567 (35%)	2510 (34%)
	50–64 years	excl	1245 (17%)	1252 (17%)
	≥65 years	excl	883 (12%)	993 (13%)
High Risk		348 (23%)	3004 (41%)	3536 (48%)
Vaccinated	Any vaccine	689 (45%)	3479 (47%)	3759 (51%)
	LAIV^b^	172 (25%)	164 (5%)	0 (0%)
Influenza	A(H1N1)pdm	212 (14%)	778 (11%)	excl
	A(H3N2)	excl	79 (1%)	1774 (24%)
	B	excl	473 (6%)	excl

^a^*Excl* excluded by design

^b^*LAIV* live attenuated influenza vaccine

**Fig. 1:**
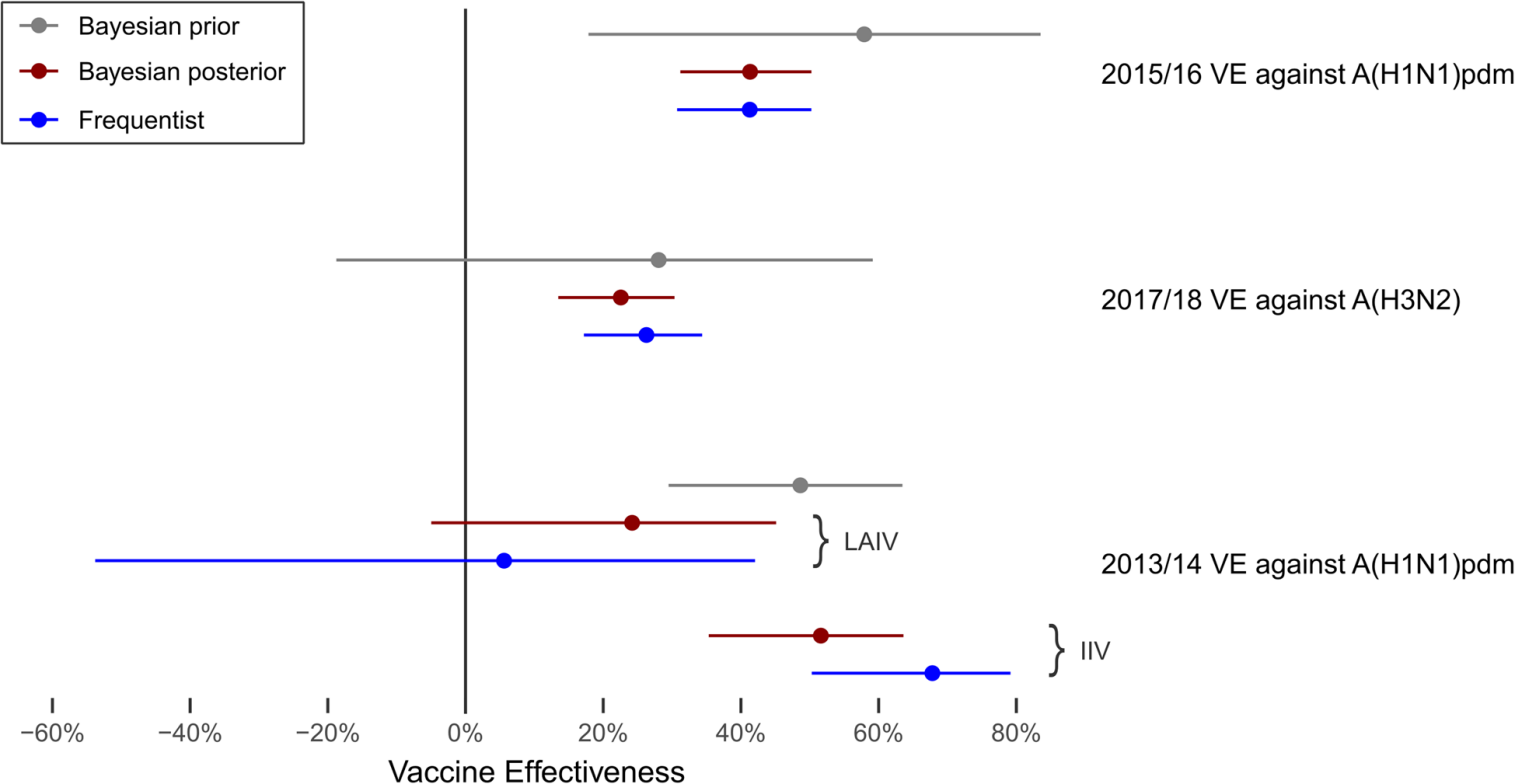
Point estimates and 95% confidence or Bayesian credible intervals for influenza vaccine effectiveness (VE) for any influenza vaccine in 2015/16 and 2017/18 and for live attenuated influenza vaccine (LAIV) and inactivated influenza vaccine (IIV) among children in 2013/14. Bayesian prior indicates estimated VE prior to collecting each season’s data, based on studies from prior seasons against the specified influenza subtype; Bayesian posterior indicates updated VE estimates after collecting the study data; Frequentist indicates estimated VE based solely on the study data without prior season information

In the 2015/16 influenza season, 6824 US Flu VE Network enrollees were eligible, of whom 3300 (48%) were vaccinated and 771 (11%) had laboratory-confirmed influenza A(H1N1)pdm infection (Table 1). After adjusting for covariates, point estimates for VE_F_ and VE_B_ against any influenza infection were virtually identical (Fig. 1). VE_F_ was 41% (95% CI, 31 to 50%), compared to VE_B_ of 41% (95% BCI, 31 to 50%).

In the 2017/18 influenza season, 7428 US Flu VE Network enrollees met the eligibility critiera, of whom 3759 (51%) were vaccinated and 1774 (24%) had laboratory-confirmed infection with influenza A(H3N2). After adjusting for covariates, point estimates for VE_F_ and VE_B_ were similar (Fig. 1). VE_F_ was 26% (95% CI, 17 to 34%), compared to VE_B_ of 23% (95% BCI, 13 to 30%).

In the 2013/14 influenza season, 1542 US Flu Network enrollees aged 2–17 years met the eligibiity criteria. Of these, 172 (25%) had been vaccinated with LAIV and 517 (33%) with IIV, while 212 (14%) were infected with laboratory-confirmed influenza A(H1N1)pdm. Estimates of LAIV effectiveness were highly divergent between the frequentist and Bayesian approaches (Fig. 1), with VE_F_ of 6% (95% CI, − 54 to 42%) compared to VE_B_ of 25% (95% BCI, − 5 to 45%). Estimates of IIV effectiveness also differed between the two approaches, with VE_F_ being higher than VE_B_ (68% vs. 52%). Under the frequentist approach, LAIV effectiveness was 62 percentage points lower than IIV, while under the Bayesian approach, LAIV effectiveness was 27 percentage points lower than IIV.

In all three analyses, Bayesian estimates with noninformative priors were nearly identical to the frequentist estimates. VE point estimates differed by no more than one percentage point, and credible interval widths were not more than one percentage point different from frequentist confidence intervals.

**Fig. 2:**
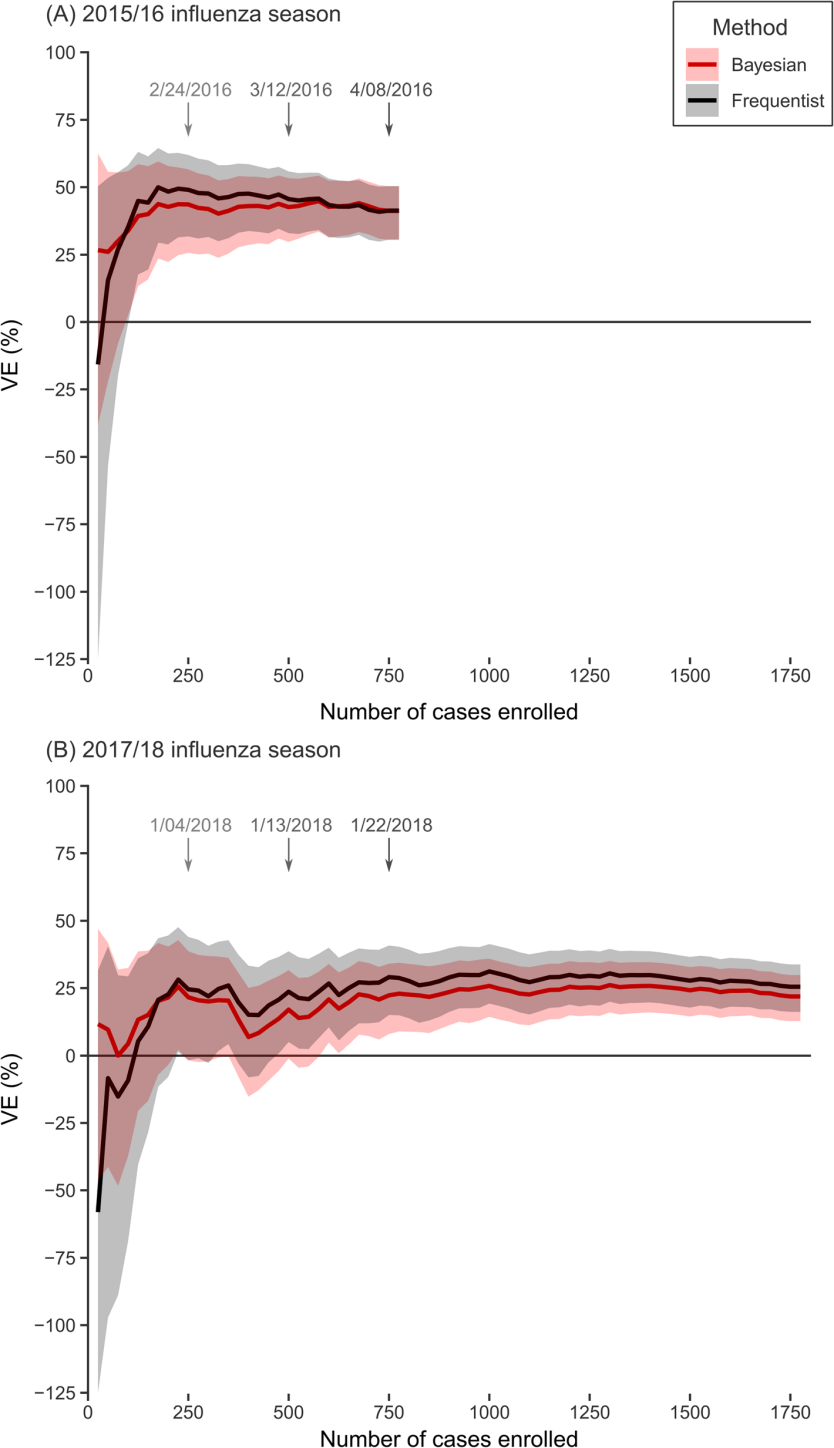
Estimated influenza vaccine effectiveness (VE) by Bayesian and frequentist methods at increasing sample size; **a** 2015/16 influenza season against A(H1N1) pdm viruses, **b** 2017/18 influenza season against A(H3N2) viruses

In 2015/16 and 2017/18, frequentist VE_F_ estimates were naturally less precise than Bayesian VE_B_ estimates early in the season (Fig. 2 for VE by cases enrolled, Supplemental Figure for VE by total enrollment). VE_F_ estimates were also more prone to rapid fluctuations. In both 2015/16 and 2017/18, VE estimates by both methods stabilized near their final value after approximately 250 cases had been enrolled. By that point, the width of the frequentist confidence interval was approximately equal to the width of the Bayesian credible interval.

## Discussion

The decision to use a frequentist vs. a Bayesian approach to estimating population parameters is ultimately a theoretical judgment about statistical inference and the nature of probability. Frequentist statistics treat probabilities as long-run frequencies, while Bayesian statistitics treat them as degrees of belief [6, 17]. As such, decisions about which approach to use in influenza vaccine surveillance should depend on the inferential goals of the study and, to a degree, be independent of expected findings or the precision of estimates. Notwithstanding, the results of this study are instructive in several ways.

First, previous influenza VE studies have generated widely varying point estimates with varying degrees of precision [18, 19]. This heterogeneity is the result both of random sampling and of true variation in VE across seasons and populations. A consequence of this heterogeneity is that Bayesian prior distributions for VE are “weak,” in the sense of not being strongly constrained to a narrow range of values. VE estimates in excess of 80%, or lower than 0% are unlikely based on prior knowledge, but there is little precision within that range. As a result, Bayesian VE estimates in this study required a comparatively large sample size (at least 250 cases enrolled) to stabilize near the final end-of-season values. While frequentist VE estimates were highly unstable at small sample sizes, the frequentist estimates stabilized at approximately the same sample size as the Bayesian estimates, with confidence intervals of comparable width to the Bayesian credible intervals. Some authors have expressed the hope that use of Bayesian statistics in pharmacoepidemiology may allow for rigorous inference at smaller sample sizes than frequentist statistics [20]. Our study suggests this is unlikely to be possible for influenza VE surveillance.

Second, in the original US Flu VE Network study in 2013/14 (using a frequentist approach), Gaglani et al. estimated IIV VE in children aged 2–17 years of 60% compared to 17% for LAIV [12]. This 43 percentage-point difference in effectiveness, which occurred immediately after quadrivalent LAIV replaced trivalent LAIV [21], precipitated discussions about the potential for LAIV-specific vaccine failure. When this finding was repeated in the 2015/16 season [1], also dominated by A(H1N1)pdm, the ACIP removed their recommendation for use of LAIV [22]. In the present analysis (with slightly simplified exclusion criteria and analyses) the frequentist estimates were similar to the findings of Gaglani et al., with a 62 percentage point difference in VE between the vaccine types. In contrast, the Bayesian VE estimates were much more similar for IIV and LAIV, only differing by 27 percentage points. It is, of course, impossible to know what would have happened had a Bayesian framework been used in 2013/14 or 2015/16. The smaller relative difference in VE_B_ may have led to the findings being explained away as a result of chance, rather than a prompt to greater vigilance regarding LAIV in children. But it also bears mentioning that the Bayesian estimate of 25% VE (− 5 to 45%) for LAIV is close to estimates from a meta-analysis of three US studies during 2013/14 (including Gaglani et al), which found a consolidated VE for LAIV of 19% (95% CI, − 18 to 44%) against A(H1N1)pdm [23]. In a situation where the data are quite different from what is expected based on prior knowledge, particularly if sample sizes are small, varying the Bayesian analysis with priors that give more weight to the data can help reveal the impact of prior assumptions on final estimates [7].

In this study, we defined Bayesian priors from observational test-negative studies of influenza VE. Other sources of data are possible, including randomized clinical trials (RCTs). RCT data are likely less subject to bias than observational data, but have important limitations as sources of prior data. Recent placebo-controlled RCTs of tri- or quadrivalent influenza vaccines are uncommon in countries with routine influenza vaccination programs, and generally predate the emergence of influenza A(H1N1)pdm in 2009. Thus, we do not have RCT data for comparable populations to the test-negative study populations. In addition, observational test-negative studies are much more common than RCTs and provide more estimates across different sub-groups defined by demographics or infecting virus. However, use of observational data may lead to systematic biases in the Bayesian priors if the test-negative studies themselves are systematically biased.

The biggest limitation of this study is conceptual. The true interest is assessing how inference about VE might differ between frequentist and Bayesian approachs for selected seasons. This is not fully possible, because the study is retrospective and frequentist VE estimates have already been disseminated, shaping our inference about VE. Nonetheless, comparing the two approaches does give some idea about when and why frequentist and Bayesian estimates are likely to be substantively different. A second limitation is the relatively few examples of truly unexpected variation in VE to use as test cases for the two methods of inference. Since the founding of the current iteration of the US Flu VE Network in 2011/12, the only situations where VE has been meaningfully lower than expectation are the low effectiveness of LAIV against A(H1N1)pdm and the low 2017/18 VE against A(H3N2) despite apparently good antigenic match. While the rarity of unexpectedly poor vaccine performance is good for public health, it does limit our ability to characterize the potentially misleading impact of prior knowledge in Bayesian VE estimation. Thirdly, we developed Bayesian prior distributions in this study using previously published VE estimates, stratified where possible by age group and virus type/subtype. Additional stratification could potentially improve the prior distributions, particularly stratifying by genetic or antigenic similarity between vaccine and circulating viruses. As more seasons of influenza VE data accumulate, our ability to more precisely define prior estimates may improve. Finally, models of VE estimates used to construct prior distributions were adjusted for varying sets of potential confounders and not necessarily the same set used in the study data. This may reduce the comparability of the priors to the study data.

## Conclusions

In this study, we have shown some of the potential impacts of Bayesian vs. frequentist reasoning in the context of influenza vaccine effectiveness surveillance. If the inferential goal is to identify potential vaccine failures, priors based on previous influenza VE studies may obscure vaccine failure in Bayesian analyses. For this goal, frequentist analyses or Bayesian analyses with “uninformative” priors may be more appropriate. If the goal is to estimate VE in small subgroups when overall VE seems consistent with expectations, Bayesian methods with informative priors may be useful. Future work may better identify situations in which informative priors aid in public health decision-making.

## Supplementary Information

**Additional file 1: Supplemental Appendix Table**. Studies for informing prior distributions for vaccine effectiveness estimates. Grey text indicates estimates not used due to being included in other publications. **Supplemental Figure**. Estimated influenza vaccine effectiveness (VE) by Bayesian and frequentist methods at increasing sample size; (A) 2015/16 influenza season, (B) 2017/18 influenza season.

## Data Availability

Data used in generating Bayesian prior distributions are contained in the Supplemental Appendix. United States Influenza Vaccine Effectiveness (US Flu VE) Network data are not publicly available, but aggregate analytic datasets are available for public health research from the corresponding author upon reasonable request.

## Abbreviations

IIV: Inactivated influenza vaccine
LAIV: Live attenuated influenza vaccine
RT-PCR: Real-time reverse transcriptase polymerase chain reaction
US Flu VE Network: United States Influenza Vaccine Effectiveness Network
VE: Vaccine effectiveness
