# Supplementary material for: Differences between Frequentist and Bayesian inference in routine surveillance for influenza vaccine effectiveness: a test-negative case-control study
Source: BMC Public Health. 2021 Mar 16;21:516. doi: 10.1186/s12889-021-10543-z (PMC7968177; doi:10.1186/s12889-021-10543-z)
Supplement: Supplementary file 1 — Additional file 1: Supplemental Appendix Table. Studies for informing prior distributions for vaccine effectiveness estimates. Grey text indicates estimates not used due to being included in other publications. Supplemental Figure. Estimated influenza vaccine effectiveness (VE) by Bayesian and frequentist methods at increasing sample size; (A) 2015/16 influenza season, (B) 2017/18 influenza season. [file 12889_2021_10543_MOESM1_ESM.docx]

**Supplemental Appendix Table:** Studies for informing prior distributions for vaccine effectiveness estimates. Grey text indicates estimates not used due to being included in other publications.

| Study | Season | Age group | Min age | Max age | Vaccine type | Type/ subtype | Sample size | Flu positive | VE | LCL | UCL |
| --- | --- | --- | --- | --- | --- | --- | --- | --- | --- | --- | --- |
| Simpson *Eurosurv* 2015[1] | 2001/02 | All ages | 0 | 99 | IIV | Any | 365 | 55 | 77% | -117% | 98% |
|  | 2002/03 | All ages | 0 | 99 | IIV | Any | 241 | 21 | 68% | -310% | 98% |
|  | 2003/04 | All ages | 0 | 99 | IIV | Any | 325 | 56 | 49% | -58% | 84% |
|  | 2004/05 | All ages | 0 | 99 | IIV | Any | 400 | 49 | 44% | -66% | 81% |
|  | 2005/06 | All ages | 0 | 99 | IIV | Any | 611 | 141 | 29% | -109% | 76% |
|  | 2006/07 | All ages | 0 | 99 | IIV | Any | 254 | 26 | 22% | -375% | 87% |
|  | 2007/08 | All ages | 0 | 99 | IIV | Any | 257 | 43 | 80% | 21% | 95% |
|  | 2008/09 | All ages | 0 | 99 | IIV | Any | 294 | 40 | 38% | -136% | 84% |
| Skowronski *Canad Comm Dis Rep* 2005[2] | 2004/05 | All ages | 0 | 99 | IIV | Any | 134 | 47 | 45% | -44% | 79% |
| Skowronski *Vaccine* 2007[3] | 2005/06 | All ages | 0 | 99 | IIV | Any | 424 | 206 | 61% | 26% | 79% |
| Kelly *PLoS One* 2009[4] | 2003 | All ages | 0 | 99 | IIV | Any | 350 | 127 | 40% | -13% | 68% |
|  | 2004 | All ages | 0 | 99 | IIV | Any | 198 | 38 | 52% | -28% | 82% |
|  | 2005 | All ages | 0 | 99 | IIV | Any | 307 | 134 | 34% | -33% | 67% |
|  | 2006 | All ages | 0 | 99 | IIV | Any | 276 | 95 | 16% | -77% | 60% |
|  | 2007 | All ages | 0 | 99 | IIV | Any | 348 | 166 | 54% | 15% | 75% |
| Skowronski *J Infect Dis* 2009[5] | 2006/07 | >=9 y | 9 | 99 | IIV | Any | 841 | 337 | 46% | 17% | 65% |
|  |  | >=9 y | 9 | 99 | IIV | A(H3N2) | 746 | 242 | 41% | 6% | 63% |
|  |  | >=9 y | 9 | 99 | IIV | B | 540 | 36 | 12% | -134% | 67% |
|  |  | 20-64 y | 20 | 64 | IIV | All | 613 | 241 | 24% | -22% | 52% |
|  |  | 20-64 y | 20 | 64 | IIV | A(H3N2) | 546 | 174 | 21% | -33% | 53% |
|  |  | 20-64 y | 20 | 64 | IIV | B | 404 | 32 | -11% | -210% | 60% |
| Janjua *J Infect Dis* 2012[6] | 2007/08 | All ages | 0 | 99 | IIV | Any | 1425 | 689 | 60% | 45% | 71% |
|  |  | All ages | 0 | 99 | IIV | A(H3N2) | 946 | 210 | 57% | 32% | 73% |
|  |  | All ages | 0 | 99 | IIV | B | 1000 | 264 | 55% | 32% | 70% |
| Fielding *BMC Infect Dis* 2011[7] | 2007 | All ages | 0 | 99 | N/S | Any | 386 | 194 | 59% | 25% | 78% |
|  |  | 20-64 y | 20 | 64 | N/S | Any | 289 | 135 | 64% | 29% | 82% |
|  |  | All ages | 0 | 99 | N/S | A(H1N1) | 371 | 49 | 27% | -92% | 72% |
|  |  | All ages | 0 | 99 | N/S | A(H3N2) | 377 | 117 | 68% | 32% | 85% |
|  |  | All ages | 0 | 99 | N/S | B | 384 | 26 | 84% | -2% | 98% |
|  | 2008 | All ages | 0 | 99 | N/S | Any | 330 | 106 | 9% | -96% | 58% |
|  |  | 20-64 y | 20 | 64 | N/S | Any | 238 | 68 | 35% | -56% | 73% |
|  |  | All ages | 0 | 99 | N/S | A(H3N2) | 325 | 38 | -66% | -349% | 39% |
| Kelly *Pediatr Infect Dis J* 2011[8] | 2008 (SH) | 6–59 m | 0 | 4 | IIV | Any | 289 | 48 | 58% | 9% | 81% |
| Kissling *Eurosurv* 2009[9] | 2008/09 | >=65 y | 65 | 99 | IIV | Any | 327 | 159 | 55% | 28% | 72% |
| Levy *Vaccine* 2014[10] | 2010 | All ages | 0 | 99 | IIV | Any | 448 | 146 | 68% | 35% | 85% |
|  |  | All ages | 0 | 99 | IIV | A(H1N1) | 385 | 83 | 84% | 53% | 94% |
|  |  | All ages | 0 | 99 | IIV | A(H3N2) | 309 | 7 | -30% | -585% | 75% |
|  |  | All ages | 0 | 99 | IIV | B | 358 | 56 | 75% | 28% | 91% |
|  |  | <5 y | 0 | 4 | IIV | Any | 307 | 10 | 1% | -209% | 68% |
|  |  | <18 y | 0 | 17 | IIV | Any | 103 | 53 | 84% | 24% | 97% |
|  |  | 18-64 y | 18 | 64 | IIV | Any | 355 | 85 | 67% | 19% | 86% |
|  | 2011 | All ages | 0 | 99 | IIV | Any | 351 | 105 | 52% | 1% | 77% |
|  |  | All ages | 0 | 99 | IIV | A(H1N1) | 315 | 69 | 71% | 15% | 90% |
|  |  | All ages | 0 | 99 | IIV | A(H3N2) | 264 | 18 | -55% | -386% | 51% |
|  |  | All ages | 0 | 99 | IIV | B | 264 | 18 | 85% | -30% | 98% |
|  |  | 18-64 y | 18 | 64 | IIV | Any | 348 | 86 | 40% | -22% | 70% |
|  | 2012 | All ages | 0 | 99 | IIV | Any | 1361 | 603 | 49% | 30% | 63% |
|  |  | All ages | 0 | 99 | IIV | A(H1N1) | 764 | 6 | 8% | -868% | 91% |
|  |  | All ages | 0 | 99 | IIV | A(H3N2) | 1090 | 332 | 46% | 21% | 63% |
|  |  | All ages | 0 | 99 | IIV | B | 1017 | 259 | 54% | 26% | 71% |
|  |  | <5 y | 0 | 4 | IIV | Any | 194 | 42 | 24% | -216% | 82% |
|  |  | <18 y | 0 | 17 | IIV | Any | 332 | 205 | 62% | 0% | 85% |
|  |  | 18-64 y | 18 | 64 | IIV | Any | 804 | 304 | 47% | 19% | 65% |
|  |  | >=65 y | 65 | 99 | IIV | Any | 130 | 55 | 62% | 15% | 83% |
| Skowronski *Clin Infect Dis* 2012[11] | 2010/11 | All ages | 0 | 99 | IIV | Any | 1718 | 709 | 37% | 17% | 52% |
|  |  | All ages | 0 | 99 | IIV | A(H1N1) | 1102 | 93 | 59% | 14% | 80% |
|  |  | All ages | 0 | 99 | IIV | A(H3N2) | 1417 | 408 | 39% | 12% | 56% |
|  |  | All ages | 0 | 99 | IIV | B | 1009 | 199 | 25% | -18% | 52% |
|  |  | <20 y | 0 | 19 | IIV | Any | 542 | 328 | 27% | -23% | 57% |
|  |  | 20-49 y | 20 | 49 | IIV | Any | 838 | 355 | 49% | 22% | 67% |
|  |  | >=50 y | 50 | 99 | IIV | Any | 338 | 116 | 26% | -28% | 57% |
|  |  | <20 y | 0 | 19 | IIV | A(H3N2) | 415 | 111 | 52% | -7% | 78% |
|  |  | 20-49 y | 20 | 49 | IIV | A(H3N2) | 699 | 216 | 39% | 0% | 63% |
|  |  | >=50 y | 50 | 99 | IIV | A(H3N2) | 304 | 82 | 34% | -25% | 65% |
|  |  | <20 y | 0 | 19 | IIV | B | 415 | 111 | -7% | -103% | 44% |
|  |  | 20-49 y | 20 | 49 | IIV | B | 552 | 69 | 66% | 10% | 87% |
|  |  | >=50 y | 50 | 99 | IIV | B | 241 | 19 | -20% | -239% | 57% |
|  |  | <20 y | 0 | 19 | IIV | A(H1N1) | 317 | 13 | 59% | -238% | 95% |
|  |  | 20-49 y | 20 | 49 | IIV | A(H1N1) | 549 | 66 | 65% | 8% | 87% |
|  |  | >=50 y | 50 | 99 | IIV | A(H1N1) | 235 | 13 | 30% | -177% | 82% |
| Pebody *Epidem Infect* 2013 | 2010/11 | All ages | 0 | 99 | IIV | A(H1N1) | 6547 | 1817 | 56% | 42% | 66% |
|  |  | All ages | 0 | 99 | IIV | B | 5941 | 1211 | 57% | 42% | 68% |
|  |  | <5 y | 0 | 4 | IIV | A(H1N1) | 648 | 146 | 87% | 45% | 97% |
|  |  | 5-14 y | 5 | 14 | IIV | A(H1N1) | 657 | 198 | 84% | 27% | 97% |
|  |  | <5 y | 0 | 4 | IIV | B | 595 | 93 | 47% | -337% | 93% |
|  |  | 5-14 y | 5 | 14 | IIV | B | 811 | 352 | 75% | 32% | 91% |
| Kissling *PLoS One* 2011[12] | 2010/11 | All ages | 0 | 99 | IIV | Any | 4410 | 2019 | 52% | 30% | 67% |
|  |  | All ages | 0 | 99 | IIV | A(H1N1) | 3344 | 1179 | 55% | 29% | 72% |
|  |  | All ages | 0 | 99 | IIV | B | 2944 | 765 | 50% | 14% | 71% |
| Fielding *Eurosurv* 2012[13] | 2011 (SH) | All ages | 0 | 99 | N/S | Any | 529 | 155 | 56% | -2% | 81% |
|  |  | <20 y | 0 | 19 | N/S | Any | 175 | 67 | 33% | -676% | 94% |
|  |  | 20-64 y | 20 | 64 | N/S | Any | 334 | 85 | 61% | -3% | 85% |
| Kelly *Med J Aust* 2016[14] | 2011 | All ages | 0 | 99 | N/S | Any | 642 | 180 | 35% | -44% | 71% |
|  | 2012 | All ages | 0 | 99 | N/S | Any | 684 | 268 | 53% | 19% | 72% |
|  | 2013 | All ages | 0 | 99 | N/S | Any | 354 | 79 | 65% | 5% | 87% |
| Castilla *Eurosurv* 2013[15] | 2011/12 | All ages | 0 | 99 | IIV | Any | 757 | 411 | 31% | -21% | 64% |
| Ohmit *Clin Infect Dis* 2013[16] | 2011/12 | All ages | 0 | 99 | N/S | Any | 4771 | 681 | 47% | 36% | 56% |
|  |  | <9 y | 0 | 8 | N/S | Any | 1490 | 190 | 45% | 20% | 62% |
|  |  | 9-17 y | 9 | 17 | N/S | Any | 666 | 111 | 58% | 27% | 76% |
|  |  | 18-49 y | 18 | 49 | N/S | Any | 1549 | 231 | 44% | 21% | 60% |
|  |  | 50-64 y | 50 | 64 | N/S | Any | 682 | 96 | 54% | 23% | 72% |
|  |  | >=65 y | 65 | 99 | N/S | Any | 384 | 53 | 43% | -18% | 72% |
|  |  | <9 y | 0 | 8 | IIV | Any | 945 | 158 | 40% | 6% | 62% |
|  |  | 9-17 y | 9 | 17 | IIV | Any | 588 | 105 | 61% | 28% | 79% |
|  |  | <9 y | 0 | 8 | LAIV | Any | 658 | 121 | 61% | 16% | 82% |
|  |  | 9-17 y | 9 | 17 | LAIV | Any | 456 | 88 | 60% | -15% | 86% |
|  |  | All ages | 0 | 99 | N/S | A(H1N1) | 4200 | 110 | 65% | 44% | 77% |
|  |  | All ages | 0 | 99 | N/S | A(H3N2) | 4530 | 440 | 39% | 23% | 52% |
|  |  | All ages | 0 | 99 | N/S | B | 4221 | 131 | 58% | 35% | 73% |
| Skowronski *Clin Infect Dis* 2014[17] | 2011/12 | All ages | 0 | 99 | IIV | Any | 1507 | 447 | 59% | 43% | 70% |
|  |  | All ages | 0 | 99 | IIV | A(H1N1) | 1143 | 83 | 80% | 52% | 92% |
|  |  | All ages | 0 | 99 | IIV | A(H3N2) | 1286 | 126 | 51% | 10% | 73% |
|  |  | All ages | 0 | 99 | IIV | B | 1292 | 232 | 51% | 26% | 67% |
|  |  | <20 y | 0 | 19 | IIV | Any | 398 | 134 | 64% | 23% | 84% |
|  |  | <20 y | 0 | 19 | IIV | A(H3N2) | 303 | 39 | 77% | -7% | 95% |
|  |  | <20 y | 0 | 19 | IIV | B | 344 | 80 | 44% | -32% | 76% |
|  |  | 20-49 y | 20 | 49 | IIV | Any | 702 | 214 | 56% | 26% | 74% |
|  |  | 20-49 y | 20 | 49 | IIV | A(H1N1) | 543 | 55 | 78% | 27% | 93% |
|  |  | 20-49 y | 20 | 49 | IIV | A(H3N2) | 549 | 61 | 44% | -41% | 78% |
|  |  | 20-49 y | 20 | 49 | IIV | B | 583 | 95 | 51% | 2% | 75% |
|  |  | >=50 y | 50 | 99 | IIV | Any | 407 | 99 | 58% | 30% | 75% |
|  |  | >=50 y | 50 | 99 | IIV | A(H1N1) | 322 | 14 | 74% | 1% | 93% |
|  |  | >=50 y | 50 | 99 | IIV | A(H3N2) | 334 | 26 | 63% | 5% | 86% |
|  |  | >=50 y | 50 | 99 | IIV | B | 365 | 57 | 50% | 7% | 73% |
| Kissling *Eurosurv* 2013[18] | 2011/12 | All ages | 0 | 99 | N/S | A(H3N2) | 1014 | 437 | 25% | -6% | 46% |
|  |  | <15 y | 0 | 14 | N/S | A(H3N2) | 78 | 38 | 19% | -170% | 76% |
|  |  | 15-59 y | 15 | 59 | N/S | A(H3N2) | 431 | 164 | 63% | 26% | 82% |
|  |  | >=60 y | 60 | 99 | N/S | A(H3N2) | 505 | 244 | 15% | -33% | 46% |
| Pebody *Eurosurv* 2013[19] | 2011/12 | All ages | 0 | 99 | IIV | A(H3N2) | 3824 | 396 | 23% | -10% | 47% |
|  |  | All ages | 0 | 99 | IIV | B | 3473 | 45 | 92% | 38% | 99% |
|  |  | <5 y | 0 | 4 | IIV | A(H3N2) | 314 | 57 | 52% | -446% | 96% |
|  |  | 5-14 y | 5 | 14 | IIV | A(H3N2) | 357 | 65 | 69% | -172% | 97% |
|  |  | 15-44 y | 15 | 44 | IIV | A(H3N2) | 1769 | 160 | 7% | -67% | 48% |
|  |  | 45-64 y | 45 | 64 | IIV | A(H3N2) | 920 | 86 | 11% | -56% | 49% |
|  |  | >=65 y | 65 | 99 | IIV | A(H3N2) | 449 | 26 | 48% | -50% | 82% |
| Sullivan *Med J Austral* 2014[20] | 2012 | All ages | 0 | 99 | N/S | Any | 1414 | 593 | 23% | -4% | 43% |
|  |  | All ages | 0 | 99 | N/S | B | 927 | 106 | 53% | 5% | 77% |
|  |  | <18 y | 0 | 17 | N/S | Any | 346 | 190 | 12% | -125% | 66% |
|  |  | 18-64 y | 18 | 64 | N/S | Any | 926 | 352 | 12% | -22% | 36% |
|  |  | >=65 y | 65 | 99 | N/S | Any | 142 | 51 | 67% | 23% | 86% |
| Sullivan *J Med Virol* 2014[21] | 2012 | All ages | 0 | 99 | N/S | Any | 600 | 239 | 45% | 8% | 66% |
|  |  | All ages | 0 | 99 | N/S | A(H3N2) | 534 | 187 | 35% | -11% | 62% |
| Kissling *Eurosurv* 2014[22] | 2012/13 | All ages | 0 | 99 | IIV | B | 4344 | 1937 | 49% | 32% | 62% |
|  |  | All ages | 0 | 99 | IIV | A(H1N1) | 3196 | 1068 | 50% | 28% | 66% |
|  |  | All ages | 0 | 99 | IIV | A(H3N2) | 3012 | 729 | 42% | 15% | 61% |
|  |  | <15 y | 0 | 14 | IIV | B | 1969 | 940 | 22% | -37% | 56% |
|  |  | <15 y | 0 | 14 | IIV | A(H1N1) | 1210 | 322 | 36% | -44% | 72% |
|  |  | <15 y | 0 | 14 | IIV | A(H3N2) | 1252 | 325 | 36% | -41% | 71% |
|  |  | 15-59 y | 15 | 59 | IIV | B | 1994 | 860 | 64% | 42% | 77% |
|  |  | 15-59 y | 15 | 59 | IIV | A(H1N1) | 1709 | 693 | 56% | 28% | 73% |
|  |  | 15-59 y | 15 | 59 | IIV | A(H3N2) | 1357 | 328 | 44% | -4% | 67% |
| McLean *J Infect Dis* 2015[23] | 2012/13 | All ages | 0 | 99 | N/S | Any | 6452 | 2307 | 49% | 43% | 55% |
|  |  | <9 y | 0 | 8 | N/S | Any | 1509 | 555 | 57% | 45% | 67% |
|  |  | 9-17 y | 9 | 17 | N/S | Any | 981 | 420 | 39% | 18% | 54% |
|  |  | 18-49 y | 18 | 49 | N/S | Any | 2267 | 739 | 39% | 26% | 50% |
|  |  | 50-64 y | 50 | 64 | N/S | Any | 1040 | 370 | 65% | 54% | 74% |
|  |  | >=65 y | 65 | 99 | N/S | Any | 655 | 223 | 26% | -10% | 50% |
|  |  | All ages | 0 | 99 | N/S | A(H3N2) | 5437 | 1292 | 39% | 29% | 47% |
|  |  | <9 y | 0 | 8 | N/S | A(H3N2) | 1196 | 242 | 51% | 32% | 64% |
|  |  | 9-17 y | 9 | 17 | N/S | A(H3N2) | 739 | 178 | 24% | -12% | 49% |
|  |  | 18-49 y | 18 | 49 | N/S | A(H3N2) | 2006 | 478 | 34% | 16% | 48% |
|  |  | 50-64 y | 50 | 64 | N/S | A(H3N2) | 898 | 228 | 52% | 33% | 65% |
|  |  | >=65 y | 65 | 99 | N/S | A(H3N2) | 598 | 166 | 11% | -41% | 43% |
| Skowronski *PLoS One* 2014[24] | 2012/13 | All ages | 0 | 99 | N/S | Any | 1501 | 652 | 50% | 33% | 63% |
|  |  | All ages | 0 | 99 | N/S | A(H1N1) | 929 | 80 | 59% | 16% | 80% |
|  |  | All ages | 0 | 99 | N/S | A(H3N2) | 1244 | 395 | 41% | 17% | 59% |
|  |  | All ages | 0 | 99 | N/S | B | 1016 | 167 | 68% | 44% | 82% |
|  |  | <20 y | 0 | 19 | N/S | Any | 384 | 202 | 87% | 65% | 95% |
|  |  | <20 y | 0 | 19 | N/S | A(H1N1) | 728 | 66 | 80% | 40% | 93% |
|  |  | <20 y | 0 | 19 | N/S | A(H3N2) | 301 | 119 | 87% | 55% | 96% |
|  |  | <20 y | 0 | 19 | N/S | B | 251 | 68 | 91% | 35% | 99% |
|  |  | 20-49 y | 20 | 49 | N/S | Any | 680 | 279 | 31% | -8% | 56% |
|  |  | 20-49 y | 20 | 49 | N/S | A(H1N1) | 451 | 50 | 56% | -17% | 84% |
|  |  | 20-49 y | 20 | 49 | N/S | A(H3N2) | 567 | 166 | 17% | -40% | 51% |
|  |  | 20-49 y | 20 | 49 | N/S | B | 459 | 58 | 32% | -60% | 71% |
|  |  | >=50 y | 50 | 99 | N/S | Any | 437 | 171 | 47% | 17% | 66% |
|  |  | >=50 y | 50 | 99 | N/S | A(H1N1) | 285 | 19 | 52% | -51% | 85% |
|  |  | >=50 y | 50 | 99 | N/S | A(H3N2) | 376 | 110 | 32% | -15% | 59% |
|  |  | >=50 y | 50 | 99 | N/S | B | 306 | 40 | 65% | 22% | 84% |
| Andrews *Eurosurv* 2014[25] | 2012/13 | All ages | 0 | 99 | IIV | A(H3N2) | 2310 | 354 | 26% | -4% | 48% |
|  |  | All ages | 0 | 99 | IIV | A(H1N1) | 1697 | 127 | 73% | 37% | 89% |
|  |  | All ages | 0 | 99 | IIV | B | 2324 | 827 | 51% | 34% | 63% |
|  |  | 5-14 y | 5 | 14 | IIV | B | 357 | 172 | 74% | 1% | 93% |
|  |  | 15-44 y | 15 | 44 | IIV | A(H3N2) | 1060 | 176 | 40% | -7% | 66% |
|  |  | 15-44 y | 15 | 44 | IIV | A(H1N1) | 958 | 74 | 83% | 28% | 96% |
|  |  | 15-44 y | 15 | 44 | IIV | B | 1225 | 341 | 68% | 46% | 82% |
|  |  | 45-64 y | 45 | 64 | IIV | A(H3N2) | 556 | 85 | 32% | -27% | 63% |
|  |  | 45-64 y | 45 | 64 | IIV | A(H1N1) | 502 | 31 | 90% | 20% | 99% |
|  |  | 45-64 y | 45 | 64 | IIV | B | 700 | 229 | 34% | -1% | 57% |
|  |  | >=65 y | 65 | 99 | IIV | A(H3N2) | 234 | 38 | -14% | -206% | 57% |
|  |  | >=65 y | 65 | 99 | IIV | B | 228 | 32 | 65% | 18% | 85% |
| Turner *Eurosurv* 2014[26] | 2013 (SH) | All ages | 0 | 99 | IIV | Any | 1495 | 482 | 56% | 34% | 70% |
|  |  | All ages | 0 | 99 | IIV | A(H1N1) | 1043 | 30 | 49% | -90% | 86% |
|  |  | All ages | 0 | 99 | IIV | A(H3N2) | 1229 | 216 | 61% | 32% | 77% |
|  |  | All ages | 0 | 99 | IIV | B | 1209 | 196 | 54% | 19% | 75% |
|  |  | <18 y | 0 | 17 | IIV | Any | 691 | 215 | 56% | 6% | 79% |
|  |  | 18-64 y | 18 | 64 | IIV | Any | 737 | 248 | 55% | 24% | 73% |
|  |  | >=65 y | 65 | 99 | IIV | Any | 67 | 19 | 76% | 15% | 93% |
| Valenciano *Vaccine* 2015[27] | 2013/14 | All ages | 0 | 99 | IIV | A(H1N1) | 2113 | 521 | 48% | 16% | 67% |
|  |  | <15 y | 0 | 14 | IIV | A(H1N1) | 698 | 110 | 65% | -86% | 93% |
|  |  | 15-59 y | 15 | 59 | IIV | A(H1N1) | 1189 | 369 | 39% | -14% | 67% |
|  |  | All ages | 0 | 99 | IIV | A(H3N2) | 2370 | 613 | 25% | -33% | 57% |
| Gaglani *J Infect Dis* 2016[28] | 2013/14 | All ages | 0 | 99 | N/S | A(H1N1) | 5462 | 4022 | 54% | 46% | 61% |
|  |  | <9 y | 0 | 8 | N/S | A(H1N1) | 1105 | 151 | 48% | 22% | 66% |
|  |  | 9-17 y | 9 | 17 | N/S | A(H1N1) | 594 | 79 | 54% | 16% | 75% |
|  |  | 18-49 y | 18 | 49 | N/S | A(H1N1) | 2099 | 476 | 53% | 39% | 64% |
|  |  | 50-64 y | 50 | 64 | N/S | A(H1N1) | 1062 | 235 | 64% | 48% | 74% |
|  |  | >=65 y | 65 | 99 | N/S | A(H1N1) | 602 | 81 | 59% | 25% | 77% |
|  |  | 2-17 y | 2 | 17 | IIV | A(H1N1) | 1295 | 182 | 60% | 36% | 74% |
|  |  | 2-17 y | 2 | 17 | LAIV | A(H1N1) | 945 | 172 | 17% | -39% | 51% |
| Skowronski *J Infect Dis* 2015[29] | 2013/14 | All ages | 0 | 99 | N/S | Any | 1700 | 663 | 68% | 58% | 76% |
|  |  | All ages | 0 | 99 | N/S | A(H1N1) | 1452 | 415 | 71% | 58% | 80% |
|  |  | <20 y | 0 | 19 | N/S | Any | 369 | 138 | 77% | 47% | 90% |
|  |  | 20-64 y | 20 | 64 | N/S | Any | 1182 | 622 | 67% | 54% | 76% |
|  |  | >=65 y | 65 | 99 | N/S | Any | 149 | 41 | 60% | 15% | 82% |
| Pierce *Vaccine* 2016[30] | 2014 | All ages | 0 | 99 | N/S | Any | 1154 | 477 | 56% | 35% | 70% |
|  |  | All ages | 0 | 99 | N/S | A(H1N1) | 1001 | 324 | 59% | 36% | 74% |
|  |  | All ages | 0 | 99 | N/S | A(H3N2) | 730 | 53 | -10% | -152% | 52% |
|  |  | All ages | 0 | 99 | N/S | B | 758 | 81 | 65% | 19% | 85% |
| Pebody *Eurosurv* 2015[31] | 2014/15 | All ages | 0 | 99 | N/S | Any | 2931 | 902 | 34% | 18% | 48% |
|  |  | All ages | 0 | 99 | N/S | A(H3N2) | 2658 | 629 | 29% | 9% | 45% |
|  |  | All ages | 0 | 99 | N/S | B | 2213 | 184 | 46% | 14% | 66% |
|  |  | <18 y | 0 | 17 | LAIV | A(H3N2) | 632 | 134 | 35% | -30% | 68% |
|  |  | <18 y | 0 | 17 | IIV | A(H3N2) | 570 | 127 | -73% | -457% | 46% |
|  |  | <18 y | 0 | 17 | LAIV | B | 527 | 30 | 100% | 17% | 100% |
|  |  | <18 y | 0 | 17 | IIV | B | 474 | 32 | -124% | -1343% | 65% |
| Valenciano *Eurosurv* 2016[32] | 2014/15 | All ages | 0 | 99 | IIV | A(H3N2) | 4491 | 1723 | 14% | -6% | 31% |
|  |  | <15 y | 0 | 14 | IIV | A(H3N2) | 1505 | 607 | 21% | -22% | 49% |
|  |  | 15-59 y | 15 | 59 | IIV | A(H3N2) | 2245 | 846 | 11% | -31% | 39% |
|  |  | >=60 y | 60 | 99 | IIV | A(H3N2) | 741 | 270 | 16% | -20% | 41% |
|  |  | All ages | 0 | 99 | IIV | A(H1N1) | 2920 | 515 | 54% | 31% | 70% |
|  |  | <15 y | 0 | 14 | IIV | A(H1N1) | 1023 | 211 | 73% | 40% | 88% |
|  |  | 15-59 y | 15 | 59 | IIV | A(H1N1) | 1436 | 245 | 60% | 11% | 82% |
|  |  | >=60 y | 60 | 99 | IIV | A(H1N1) | 451 | 59 | 22% | -44% | 58% |
|  |  | All ages | 0 | 99 | IIV | B | 3730 | 1001 | 48% | 29% | 62% |
|  |  | <15 y | 0 | 14 | IIV | B | 1143 | 269 | 62% | 15% | 83% |
|  |  | 15-59 y | 15 | 59 | IIV | B | 1986 | 602 | 41% | 6% | 63% |
|  |  | >=60 y | 60 | 99 | IIV | B | 601 | 130 | 50% | 15% | 71% |
| Zimmerman *Clin Infect Dis* 2016[33] | 2014/15 | All ages | 0 | 99 | N/S | Any | 9311 | 2233 | 22% | 13% | 30% |
|  |  | <9 y | 0 | 8 | N/S | Any | 2419 | 473 | 26% | 7% | 41% |
|  |  | 9-17 y | 9 | 17 | N/S | Any | 1342 | 392 | 26% | 3% | 44% |
|  |  | 18-49 y | 18 | 49 | N/S | Any | 2848 | 642 | 9% | -11% | 26% |
|  |  | 50-64 y | 50 | 64 | N/S | Any | 1496 | 378 | 25% | 2% | 42% |
|  |  | >=65 y | 65 | 99 | N/S | Any | 1206 | 274 | 33% | 3% | 54% |
|  |  | All ages | 0 | 99 | N/S | A(H3N2) | 8895 | 1817 | 11% | -1% | 21% |
|  |  | <9 y | 0 | 8 | N/S | A(H3N2) | 2342 | 396 | 23% | 1% | 40% |
|  |  | 9-17 y | 9 | 17 | N/S | A(H3N2) | 1256 | 306 | 7% | -26% | 32% |
|  |  | 18-49 y | 18 | 49 | N/S | A(H3N2) | 2737 | 531 | -3% | -28% | 18% |
|  |  | 50-64 y | 50 | 64 | N/S | A(H3N2) | 1399 | 281 | 18% | -13% | 40% |
|  |  | >=65 y | 65 | 99 | N/S | A(H3N2) | 1206 | 303 | 15% | -28% | 43% |
| Skowronski *Clin Infect Dis* 2016[34] | 2014/15 | All ages | 0 | 99 | N/S | Any | 1930 | 815 | 9% | -14% | 27% |
|  |  | All ages | 0 | 99 | N/S | A(H3N2) | 1685 | 570 | -17% | -50% | 9% |
|  |  | All ages | 0 | 99 | N/S | B | 1341 | 226 | 45% | 18% | 64% |
|  |  | <20 y | 0 | 19 | N/S | Any | 466 | 199 | -5% | -71% | 35% |
|  |  | 20-64 y | 20 | 64 | N/S | Any | 1212 | 503 | 7% | -20% | 28% |
|  |  | >=65 y | 65 | 99 | N/S | Any | 252 | 113 | 20% | -47% | 57% |
| Yaron-Yakoby *Eurosurv* 2018[35] | 2014/15 | All ages | 0 | 99 | IIV | Any | 1005 | 307 | -5% | -55% | 29% |
|  |  | All ages | 0 | 99 | IIV | A(H3N2) | 955 | 257 | -16% | -73% | 22% |
|  |  | <18 y | 0 | 17 | IIV | Any | 588 | 175 | 31% | -18% | 59% |
|  |  | <18 y | 0 | 17 | IIV | A(H3N2) | 570 | 157 | 23% | -32% | 55% |
|  |  | >=18 y | 18 | 99 | IIV | Any | 417 | 132 | -54% | -167% | 11% |
|  |  | >=18 y | 18 | 99 | IIV | A(H3N2) | 385 | 100 | -76% | -216% | 2% |
|  | 2015/16 | All ages | 0 | 99 | IIV | Any | 1658 | 796 | 9% | -25% | 34% |
|  |  | All ages | 0 | 99 | IIV | A(H1N1) | 1216 | 332 | 32% | -4%% | 56% |
|  |  | All ages | 0 | 99 | IIV | B | 1321 | 448 | -2% | -47% | 29% |
|  |  | <18 y | 0 | 17 | IIV | Any | 815 | 371 | -25% | -98% | 21% |
|  |  | <18 y | 0 | 17 | IIV | A(H1N1) | 579 | 129 | -8% | -104% | 43% |
|  |  | <18 y | 0 | 17 | IIV | B | 692 | 242 | -25% | -107% | 25% |
|  |  | >=18 y | 18 | 99 | IIV | Any | 843 | 425 | 39% | 8% | 60% |
|  |  | >=18 y | 18 | 99 | IIV | A(H1N1) | 637 | 219 | 57% | 24% | 75% |
|  |  | >=18 y | 18 | 99 | IIV | B | 629 | 206 | 26% | -21% | 55% |
| Fielding *Vaccine* 2016[36] | 2015 | All ages | 0 | 99 | N/S | Any | 2443 | 857 | 54% | 42% | 63% |
|  |  | <18 y | 0 | 17 | N/S | Any | 634 | 273 | 68% | 33% | 84% |
|  |  | 18-64 y | 18 | 64 | N/S | Any | 1492 | 505 | 52% | 37% | 63% |
|  |  | >=65 y | 65 | 99 | N/S | Any | 317 | 79 | 51% | 10% | 73% |
|  |  | All ages | 0 | 99 | N/S | A(H1N1) | 1616 | 30 | 79% | 33% | 93% |
|  |  | All ages | 0 | 99 | N/S | A(H3N2) | 1853 | 267 | 44% | 21% | 60% |
|  |  | <18 y | 0 | 17 | N/S | A(H3N2) | 431 | 70 | 55% | -35% | 85% |
|  |  | 18-64 y | 18 | 64 | N/S | A(H3N2) | 1144 | 157 | 43% | 14% | 62% |
|  |  | >=65 y | 65 | 99 | N/S | A(H3N2) | 278 | 40 | 38% | -40% | 72% |
|  |  | All ages | 0 | 99 | N/S | B | 2133 | 547 | 58% | 45% | 68% |
|  |  | <18 y | 0 | 17 | N/S | B | 554 | 193 | 71% | 32% | 88% |
|  |  | 18-64 y | 18 | 64 | N/S | B | 1307 | 320 | 55% | 38% | 68% |
|  |  | >=65 y | 65 | 99 | N/S | B | 272 | 34 | 64% | 19% | 84% |
| Pebody *Eurosurv* 2016[37] | 2015/16 | All ages | 0 | 99 | All | Any | 3841 | 1155 | 52% | 41% | 62% |
|  |  | All ages | 0 | 99 | All | A(H1N1) | 3456 | 770 | 55% | 42% | 65% |
|  |  | All ages | 0 | 99 | All | B | 3037 | 351 | 54% | 33% | 69% |
|  |  | 2-17 y | 2 | 17 | IIV | Any | 633 | 215 | 78% | 7% | 94% |
|  |  | 2-17 y | 2 | 17 | IIV | A(H1N1) | 530 | 112 | 100% | 13% | 100% |
|  |  | 2-17 y | 2 | 17 | IIV | B | 516 | 98 | 56% | -122% | 91% |
|  |  | 2-17 y | 2 | 17 | LAIV | Any | 729 | 238 | 58% | 25% | 76% |
|  |  | 2-17 y | 2 | 17 | LAIV | A(H1N1) | 625 | 132 | 41% | -8% | 68% |
|  |  | 2-17 y | 2 | 17 | LAIV | B | 590 | 99 | 81% | 40% | 94% |
|  |  | 18-44 y | 18 | 44 | IIV | Any | 1552 | 525 | 55% | 34% | 70% |
|  |  | 18-44 y | 18 | 44 | IIV | A(H1N1) | 1370 | 348 | 60% | 35% | 75% |
|  |  | 18-44 y | 18 | 44 | IIV | B | 1192 | 170 | 46% | 1% | 70% |
|  |  | 45-64 y | 45 | 64 | IIV | Any | 908 | 258 | 55% | 35% | 70% |
|  |  | 45-64 y | 45 | 64 | IIV | A(H1N1) | 847 | 211 | 59% | 37% | 73% |
|  |  | 45-64 y | 45 | 64 | IIV | B | 683 | 47 | 65% | 15% | 86% |
|  |  | >=65 y | 65 | 99 | IIV | Any | 409 | 58 | 29% | -34% | 62% |
|  |  | >=65 y | 65 | 99 | IIV | A(H1N1) | 385 | 39 | 56% | 7% | 79% |
|  |  | >=65 y | 65 | 99 | IIV | B | 365 | 19 | -20% | -259% | 60% |
| Skowronski *J Infect Dis* 2017[38] | 2015/16 | All ages | 0 | 99 | N/S | Any | 2008 | 1082 | 46% | 32% | 57% |
|  |  | All ages | 0 | 99 | N/S | A(H1N1) | 1522 | 596 | 43% | 25% | 57% |
|  |  | All ages | 0 | 99 | N/S | B | 1349 | 423 | 50% | 31% | 63% |
| Jackson *NEJM* 2017[39] | 2015/16 | All ages | 0 | 99 | N/S | Any | 6879 | 1309 | 48% | 41% | 55% |
|  |  | <9 y | 0 | 8 | N/S | Any | 1526 | 254 | 51% | 33% | 64% |
|  |  | 9-17 y | 9 | 17 | N/S | Any | 858 | 164 | 59% | 36% | 74% |
|  |  | 18-49 y | 18 | 49 | N/S | Any | 2456 | 499 | 52% | 39% | 61% |
|  |  | 50-64 y | 50 | 64 | N/S | Any | 1201 | 283 | 26% | 2% | 44% |
|  |  | >=65 y | 65 | 99 | N/S | Any | 838 | 109 | 42% | 6% | 64% |
|  |  | 2-17 y | 2 | 17 | IIV | Any | 1908 | 357 | 60% | 47% | 70% |
|  |  | 2-17 y | 2 | 17 | LAIV | Any | 1362 | 319 | 5% | -47% | 39% |
|  |  | All ages | 0 | 99 | N/S | A(H1N1) | 6338 | 768 | 45% | 34% | 53% |
|  |  | <9 y | 0 | 8 | N/S | A(H1N1) | 1432 | 160 | 49% | 26% | 65% |
|  |  | 9-17 y | 9 | 17 | N/S | A(H1N1) | 745 | 51 | 63% | 22% | 82% |
|  |  | 18-49 y | 18 | 49 | N/S | A(H1N1) | 2268 | 311 | 46% | 29% | 59% |
|  |  | 50-64 y | 50 | 64 | N/S | A(H1N1) | 1112 | 194 | 10% | -26% | 36% |
|  |  | >=65 y | 65 | 99 | N/S | A(H1N1) | 781 | 52 | 66% | 36% | 81% |
|  |  | All ages | 0 | 99 | N/S | B | 6026 | 456 | 55% | 44% | 64% |
|  |  | <9 y | 0 | 8 | N/S | B | 1361 | 89 | 50% | 17% | 70% |
|  |  | 9-17 y | 9 | 17 | N/S | B | 801 | 107 | 53% | 19% | 73% |
|  |  | 18-49 y | 18 | 49 | N/S | B | 2102 | 145 | 68% | 51% | 79% |
|  |  | 50-64 y | 50 | 64 | N/S | B | 991 | 73 | 48% | 14% | 69% |
|  |  | >=65 y | 65 | 99 | N/S | B | 771 | 42 | -34% | -236% | 47% |
| Pebody *Eurosurv* 2017[40] | 2016/17 | 2-17 y | 2 | 17 | LAIV | Any | 567 | 123 | 66% | 30% | 83% |
|  |  | 2-17 y | 2 | 17 | IIV | Any | 465 | 112 | 43% | -184% | 89% |
|  |  | 2-17 y | 2 | 17 | LAIV | A(H3N2) | 545 | 100 | 57% | 8% | 80% |
|  |  | 2-17 y | 2 | 17 | IIV | A(H3N2) | 444 | 91 | 25% | -296% | 86% |
|  |  | All ages | 0 | 99 | All | Any | 2881 | 659 | 40% | 23% | 53% |
|  |  | All ages | 0 | 99 | All | A(H3N2) | 2746 | 514 | 32% | 10% | 48% |
|  |  | All ages | 0 | 99 | All | B | 2292 | 70 | 55% | 11% | 77% |
|  |  | 18-64 y | 18 | 64 | All | Any | 1736 | 428 | 41% | 19% | 56% |
|  |  | 18-64 y | 18 | 64 | All | A(H3N2) | 1634 | 315 | 37% | 10% | 55% |
|  |  | 18-64 y | 18 | 64 | All | B | 1351 | 43 | 52% | -20% | 81% |
|  |  | >=65 y | 65 | 99 | All | Any | 398 | 85 | -6% | -95% | 42% |
|  |  | >=65 y | 65 | 99 | All | A(H3N2) | 380 | 67 | -68% | -249% | 19% |
|  |  | >=65 y | 65 | 99 | All | B | 325 | 12 | 17% | -250% | 80% |
| Flannery *Clin Infect Dis* 2019[41] | 2016/17 | All ages | 0 | 99 | N/S | Any | 7083 | 2043 | 40% | 32% | 46% |
|  |  | <9 y | 0 | 8 | N/S | Any | 1519 | 338 | 57% | 43% | 68% |
|  |  | 9-17 y | 9 | 17 | N/S | Any | 1011 | 403 | 36% | 15% | 52% |
|  |  | 18-49 y | 18 | 49 | N/S | Any | 2165 | 528 | 19% | 0% | 34% |
|  |  | 50-64 y | 50 | 64 | N/S | Any | 1362 | 447 | 40% | 24% | 53% |
|  |  | >=65 y | 65 | 99 | N/S | Any | 1026 | 327 | 20% | -11% | 43% |
|  |  | All ages | 0 | 99 | N/S | A(H3N2) | 6382 | 1342 | 33% | 23% | 41% |
|  |  | <9 y | 0 | 8 | N/S | A(H3N2) | 1371 | 190 | 49% | 28% | 64% |
|  |  | 9-17 y | 9 | 17 | N/S | A(H3N2) | 867 | 259 | 33% | 7% | 52% |
|  |  | 18-49 y | 18 | 49 | N/S | A(H3N2) | 1988 | 351 | 13% | -11% | 32% |
|  |  | 50-64 y | 50 | 64 | N/S | A(H3N2) | 1219 | 304 | 31% | 9% | 47% |
|  |  | >=65 y | 65 | 99 | N/S | A(H3N2) | 937 | 238 | 21% | -15% | 45% |
|  |  | All ages | 0 | 99 | N/S | B | 5688 | 648 | 53% | 43% | 61% |
| Kissling *Eurosurv* 2016[42] | 2011/12 | All ages | 0 | 99 | IIV | A(H3N2) | 3876 | 1751 | 11.3% | -16% | 32% |
|  | 2012/13 | All ages | 0 | 99 | IIV | A(H3N2) | 3012 | 672 | 42% | 15% | 61% |
|  | 2013/14 | All ages | 0 | 99 | IIV | A(H3N2) | 2351 | 614 | 6% | -35% | 35% |
|  | 2014/15 | All ages | 0 | 99 | IIV | A(H3N2) | 4269 | 1722 | 15% | -6% | 31% |
|  | 2011/12 | >=60 y | 60 | 99 | IIV | A(H3N2) | 519 | 251 | 15% | -33% | 46% |
|  | 2012/13 | >=60 y | 60 | 99 | IIV | A(H3N2) | 262 | 72 | 53% | 5% | 77% |
|  | 2013/14 | >=60 y | 60 | 99 | IIV | A(H3N2) | 261 | 78 | 41% | -18% | 70% |
|  | 2014/15 | >=60 y | 60 | 99 | IIV | A(H3N2) | 708 | 270 | 15% | -20% | 40% |
|  | 2010/11 | All ages | 0 | 99 | IIV | A(H1N1) | 3255 | 1139 | 54% | 30% | 69% |
|  | 2012/13 | All ages | 0 | 99 | IIV | A(H1N1) | 3196 | 978 | 50% | 28% | 66% |
|  | 2013/14 | All ages | 0 | 99 | IIV | A(H1N1) | 2113 | 521 | 48% | 16% | 67% |
|  | 2014/15 | All ages | 0 | 99 | IIV | A(H1N1) | 2715 | 514 | 53% | 30% | 69% |
|  | 2010/11 | >=60 y | 60 | 99 | IIV | A(H1N1) | 334 | 50 | 73% | 45% | 87% |
|  | 2012/13 | >=60 y | 60 | 99 | IIV | A(H1N1) | 254 | 50 | 59% | 14% | 81% |
|  | 2013/14 | >=60 y | 60 | 99 | IIV | A(H1N1) | 226 | 42 | 52% | -1% | 77% |
|  | 2014/15 | >=60 y | 60 | 99 | IIV | A(H1N1) | 1228 | 59 | 22% | -44% | 58% |
|  | 2010/11 | All ages | 0 | 99 | IIV | B | 2885 | 754 | 55% | 27% | 72% |
|  | 2012/13 | All ages | 0 | 99 | IIV | B | 4344 | 1890 | 49% | 32% | 62% |
|  | 2014/15 | All ages | 0 | 99 | IIV | B | 3,580 | 1002 | 48% | 28% | 62% |
|  | 2011/12 | >=60 y | 60 | 99 | IIV | B | 333 | 49 | 43% | -12% | 71% |
|  | 2012/13 | >=60 y | 60 | 99 | IIV | B | 356 | 131 | 40% | -3% | 65% |
|  | 2013/14 | >=60 y | 60 | 99 | IIV | B | 570 | 129 | 53% | 19% | 73% |

N/S = vaccine type not specified

IIV may include adjuvanted vaccines in Europe

**Supplemental Figure**: Estimated influenza vaccine effectiveness (VE) by Bayesian and frequentist methods at increasing sample size; (A) 2015/16 influenza season, (B) 2017/18 influenza season


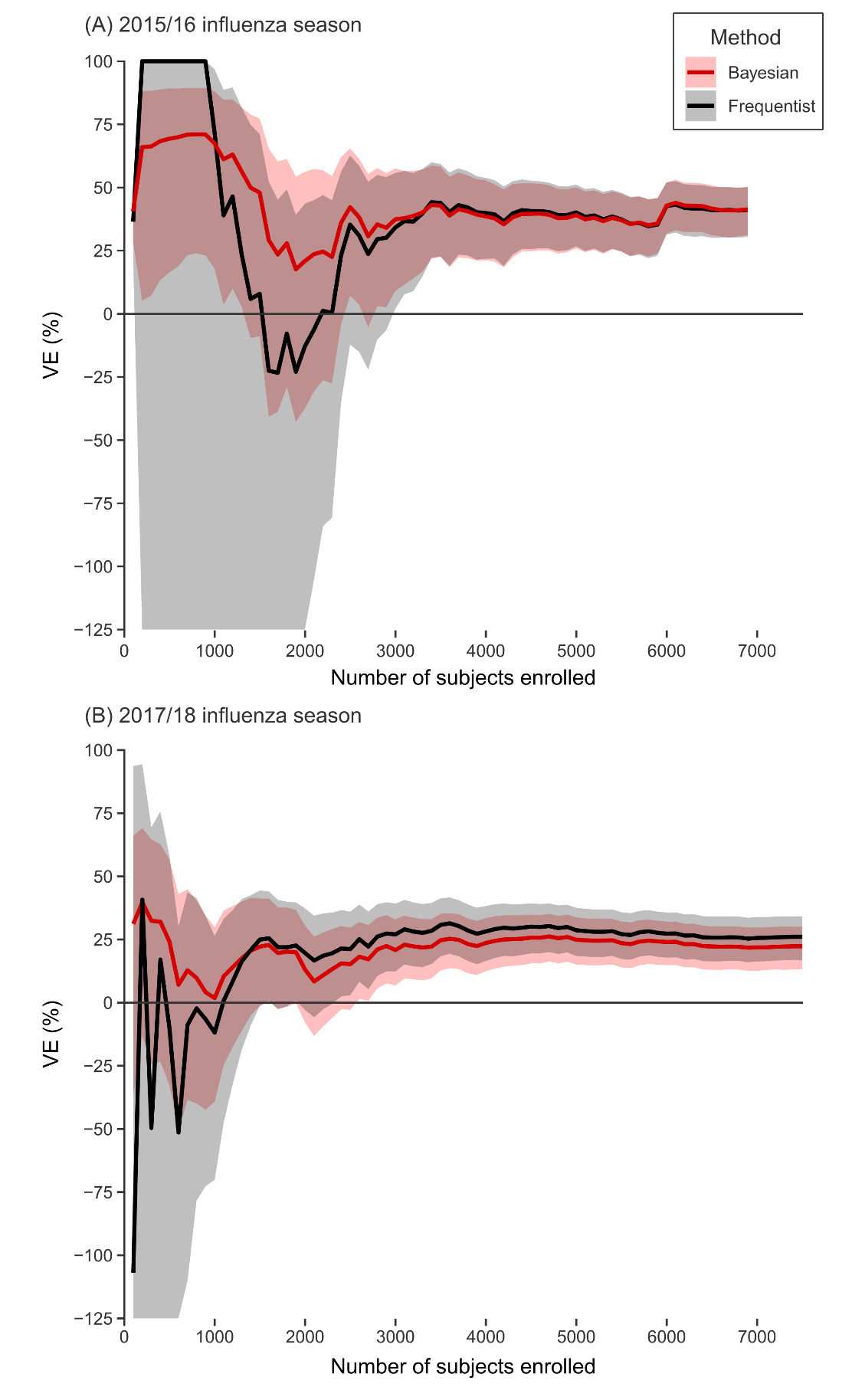


**References**

1. Simpson CR, Lone NI, Kavanagh K, Ritchie LD, Robertson C, Sheikh A, et al. Trivalent inactivated seasonal influenza vaccine effectiveness for the prevention of laboratory-confirmed influenza in a Scottish population 2000 to 2009. Euro Surveill. 2015;20(8). PubMed PMID: 25742433.

2. Skowronski DM, Gilbert M, Tweed SA, Petric M, Li Y, Mak A, et al. Effectiveness of vaccine against medical consultation due to laboratory-confirmed influenza: results from a sentinel physician pilot project in British Columbia, 2004-2005. Canada Communicable Disease Rep. 2005;31(18):181-91.

3. Skowronski DM, Masaro C, Kwindt TL, Mak A, Petric M, Li Y, et al. Estimating vaccine effectiveness against laboratory-confirmed influenza using a sentinel physician network: results from the 2005-2006 season of dual A and B vaccine mismatch in Canada. Vaccine. 2007;25(15):2842-51. doi: 10.1016/j.vaccine.2006.10.002. PubMed PMID: 17081662.

4. Kelly H, Carville K, Grant K, Jacoby P, Tran T, Barr I. Estimation of influenza vaccine effectiveness from routine surveillance data. PLoS One. 2009;4(3):e5079. doi: 10.1371/journal.pone.0005079. PubMed PMID: 19333374; PubMed Central PMCID: PMCPMC2658741.

5. Skowronski DM, De Serres G, Dickinson J, Petric M, Mak A, Fonseca K, et al. Component-specific effectiveness of trivalent influenza vaccine as monitored through a sentinel surveillance network in Canada, 2006-2007. J Infect Dis. 2009;199(2):168-79. doi: 10.1086/595862. PubMed PMID: 19086914.

6. Janjua NZ, Skowronski DM, De Serres G, Dickinson J, Crowcroft NS, Taylor M, et al. Estimates of influenza vaccine effectiveness for 2007-2008 from Canada's sentinel surveillance system: cross-protection against major and minor variants. J Infect Dis. 2012;205(12):1858-68. doi: 10.1093/infdis/jis283. PubMed PMID: 22492921.

7. Fielding JE, Grant KA, Papadakis G, Kelly HA. Estimation of type- and subtype-specific influenza vaccine effectiveness in Victoria, Australia using a test negative case control method, 2007-2008. BMC Infect Dis. 2011;11:170. doi: 10.1186/1471-2334-11-170. PubMed PMID: 21669006; PubMed Central PMCID: PMCPMC3131256.

8. Kelly H, Jacoby P, Dixon GA, Carcione D, Williams S, Moore HC, et al. Vaccine Effectiveness Against Laboratory-confirmed Influenza in Healthy Young Children: A Case-Control Study. Pediatr Infect Dis J. 2011;30(2):107-11. Epub 2010/11/17. doi: 10.1097/INF.0b013e318201811c. PubMed PMID: 21079528.

9. Kissling E, Valenciano M, Falcao J, Larrauri A, Widgren K, Pitigoi D, et al. "I-MOVE" towards monitoring seasonal and pandemic influenza vaccine effectiveness: lessons learnt from a pilot multi-centric case-control study in Europe, 2008-9. Euro Surveill. 2009;14(44). PubMed PMID: 19941774.

10. Levy A, Sullivan SG, Tempone SS, Wong KL, Regan AK, Dowse GK, et al. Influenza vaccine effectiveness estimates for Western Australia during a period of vaccine and virus strain stability, 2010 to 2012. Vaccine. 2014;32(47):6312-8. doi: 10.1016/j.vaccine.2014.08.066. PubMed PMID: 25223268.

11. Skowronski DM, Janjua NZ, De Serres G, Winter AL, Dickinson JA, Gardy JL, et al. A sentinel platform to evaluate influenza vaccine effectiveness and new variant circulation, Canada 2010-2011 season. Clin Infect Dis. 2012;55(3):332-42. doi: 10.1093/cid/cis431. PubMed PMID: 22539661.

12. Kissling E, Valenciano M, Cohen JM, Oroszi B, Barret AS, Rizzo C, et al. I-MOVE multi-centre case control study 2010-11: overall and stratified estimates of influenza vaccine effectiveness in Europe. PLoS ONE. 2011;6(11):e27622. Epub 2011/11/24. doi: 10.1371/journal.pone.0027622. PubMed PMID: 22110695; PubMed Central PMCID: PMC3216983.

13. Fielding JE, Grant KA, Tran T, Kelly HA. Moderate influenza vaccine effectiveness in Victoria, Australia, 2011. Euro Surveill. 2012;17(11). Epub 2012/03/28. PubMed PMID: 22449867.

14. Kelly HA, Lane C, Cheng AC. Influenza vaccine effectiveness in general practice and in hospital patients in Victoria, 2011-2013. Med J Aust. 2016;204(2):76 e1-4. PubMed PMID: 26821109.

15. Castilla J, Martinez-Baz I, Martinez-Artola V, Reina G, Pozo F, Garcia Cenoz M, et al. Decline in influenza vaccine effectiveness with time after vaccination, Navarre, Spain, season 2011/12. Euro Surveill. 2013;18(5). Epub 2013/02/13. PubMed PMID: 23399423.

16. Ohmit SE, Thompson MG, Petrie JG, Thaker SN, Jackson ML, Belongia EA, et al. Influenza Vaccine Effectiveness in the 2011-2012 Season: Protection Against Each Circulating Virus and the Effect of Prior Vaccination on Estimates. Clin Infect Dis. 2013;58(3):319-27. Epub 2013/11/16. doi: 10.1093/cid/cit736. PubMed PMID: 24235265.

17. Skowronski DM, Janjua NZ, Sabaiduc S, De Serres G, Winter AL, Gubbay JB, et al. Influenza A/subtype and B/lineage effectiveness estimates for the 2011-2012 trivalent vaccine: cross-season and cross-lineage protection with unchanged vaccine. J Infect Dis. 2014;210(1):126-37. doi: 10.1093/infdis/jiu048. PubMed PMID: 24446529.

18. Kissling E, Valenciano M, Larrauri A, Oroszi B, Cohen JM, Nunes B, et al. Low and decreasing vaccine effectiveness against influenza A(H3) in 2011/12 among vaccination target groups in Europe: results from the I-MOVE multicentre case-control study. Euro Surveill. 2013;18(5):33-42. Epub 2013/02/13. PubMed PMID: 23399425.

19. Pebody R, Andrews N, McMenamin J, Durnall H, Ellis J, Thompson CI, et al. Vaccine effectiveness of 2011/12 trivalent seasonal influenza vaccine in preventing laboratory-confirmed influenza in primary care in the United Kingdom: evidence of waning intra-seasonal protection. Euro Surveill. 2013;18(5). Epub 2013/02/13. PubMed PMID: 23399424.

20. Sullivan SG, Chilver MB, Higgins G, Cheng AC, Stocks NP. Influenza vaccine effectiveness in Australia: results from the Australian Sentinel Practices Research Network. Med J Aust. 2014;201(2):109-11. PubMed PMID: 25045991.

21. Sullivan SG, Komadina N, Grant K, Jelley L, Papadakis G, Kelly H. Influenza vaccine effectiveness during the 2012 influenza season in Victoria, Australia: influences of waning immunity and vaccine match. J Med Virol. 2014;86(6):1017-25. doi: 10.1002/jmv.23847. PubMed PMID: 24395730.

22. Kissling E, Valenciano M, Buchholz U, Larrauri A, Cohen JM, Nunes B, et al. Influenza vaccine effectiveness estimates in Europe in a season with three influenza type/subtypes circulating: the I-MOVE multicentre case-control study, influenza season 2012/13. Euro Surveill. 2014;19(6). Epub 2014/02/22. PubMed PMID: 24556348.

23. McLean HQ, Thompson MG, Sundaram ME, Kieke BA, Gaglani M, Murthy K, et al. Influenza vaccine effectiveness in the United States during 2012-2013: variable protection by age and virus type. J Infect Dis. 2015;211(10):1529-40. Epub 2014/11/20. doi: 10.1093/infdis/jiu647. PubMed PMID: 25406334; PubMed Central PMCID: PMC4407759.

24. Skowronski DM, Janjua NZ, De Serres G, Sabaiduc S, Eshaghi A, Dickinson JA, et al. Low 2012-13 influenza vaccine effectiveness associated with mutation in the egg-adapted H3N2 vaccine strain not antigenic drift in circulating viruses. PLoS One. 2014;9(3):e92153. doi: 10.1371/journal.pone.0092153. PubMed PMID: 24667168; PubMed Central PMCID: PMCPMC3965421.

25. Andrews N, McMenamin J, Durnall H, Ellis J, Lackenby A, Robertson C, et al. Effectiveness of trivalent seasonal influenza vaccine in preventing laboratory-confirmed influenza in primary care in the United Kingdom: 2012/13 end of season results. Euro Surveill. 2014;19(27):5-13. PubMed PMID: 25033051.

26. Turner N, Pierse N, Bissielo A, Huang Q, Radke S, Baker M, et al. Effectiveness of seasonal trivalent inactivated influenza vaccine in preventing influenza hospitalisations and primary care visits in Auckland, New Zealand, in 2013. Euro Surveill. 2014;19(34). PubMed PMID: 25188614; PubMed Central PMCID: PMCPMC4627593.

27. Valenciano M, Kissling E, Reuss A, Jimenez-Jorge S, Horvath JK, Donnell JM, et al. The European I-MOVE Multicentre 2013-2014 Case-Control Study. Homogeneous moderate influenza vaccine effectiveness against A(H1N1)pdm09 and heterogenous results by country against A(H3N2). Vaccine. 2015;33(24):2813-22. doi: 10.1016/j.vaccine.2015.04.012. PubMed PMID: 25936723.

28. Gaglani M, Pruszynski J, Murthy K, Clipper L, Robertson A, Reis M, et al. Influenza Vaccine Effectiveness Against 2009 Pandemic Influenza A(H1N1) Virus Differed by Vaccine Type During 2013-2014 in the United States. J Infect Dis. 2016;213(10):1546-56. doi: 10.1093/infdis/jiv577. PubMed PMID: 26743842; PubMed Central PMCID: PMCPMC4837903.

29. Skowronski DM, Chambers C, Sabaiduc S, De Serres G, Winter AL, Dickinson JA, et al. Integrated Sentinel Surveillance Linking Genetic, Antigenic, and Epidemiologic Monitoring of Influenza Vaccine-Virus Relatedness and Effectiveness During the 2013-2014 Influenza Season. J Infect Dis. 2015;212(5):726-39. doi: 10.1093/infdis/jiv177. PubMed PMID: 25784728.

30. Pierse N, Kelly H, Thompson MG, Bissielo A, Radke S, Huang QS, et al. Influenza vaccine effectiveness for hospital and community patients using control groups with and without non-influenza respiratory viruses detected, Auckland, New Zealand 2014. Vaccine. 2016;34(4):503-9. doi: 10.1016/j.vaccine.2015.11.073. PubMed PMID: 26685091.

31. Pebody R, Warburton F, Andrews N, Ellis J, von Wissmann B, Robertson C, et al. Effectiveness of seasonal influenza vaccine in preventing laboratory-confirmed influenza in primary care in the United Kingdom: 2014/15 end of season results. Euro Surveill. 2015;20(36). doi: 10.2807/1560-7917.ES.2015.20.36.30013. PubMed PMID: 26535911.

32. Valenciano M, Kissling E, Reuss A, Rizzo C, Gherasim A, Horvath JK, et al. Vaccine effectiveness in preventing laboratory-confirmed influenza in primary care patients in a season of co-circulation of influenza A(H1N1)pdm09, B and drifted A(H3N2), I-MOVE Multicentre Case-Control Study, Europe 2014/15. Euro Surveill. 2016;21(7):pii=30139. doi: 10.2807/1560-7917.ES.2016.21.7.30139. PubMed PMID: 26924024.

33. Zimmerman RK, Nowalk MP, Chung J, Jackson ML, Jackson LA, Petrie JG, et al. 2014-2015 Influenza Vaccine Effectiveness in the United States by Vaccine Type. Clin Infect Dis. 2016;63(12):1564-73. doi: 10.1093/cid/ciw635. PubMed PMID: 27702768; PubMed Central PMCID: PMCPMC5146719.

34. Skowronski DM, Chambers C, Sabaiduc S, De Serres G, Winter AL, Dickinson JA, et al. A Perfect Storm: Impact of Genomic Variation and Serial Vaccination on Low Influenza Vaccine Effectiveness During the 2014-2015 Season. Clin Infect Dis. 2016;63(1):21-32. doi: 10.1093/cid/ciw176. PubMed PMID: 27025838; PubMed Central PMCID: PMCPMC4901864.

35. Yaron-Yakoby H, Sefty H, Pando R, Dichtiar R, Katz MA, Stein Y, et al. Effectiveness of influenza vaccine in preventing medically-attended influenza virus infection in primary care, Israel, influenza seasons 2014/15 and 2015/16. Euro Surveill. 2018;23(7). doi: 10.2807/1560-7917.ES.2018.23.7.17-00026. PubMed PMID: 29471622; PubMed Central PMCID: PMCPMC5824129.

36. Fielding JE, Levy A, Chilver MB, Deng YM, Regan AK, Grant KA, et al. Effectiveness of seasonal influenza vaccine in Australia, 2015: An epidemiological, antigenic and phylogenetic assessment. Vaccine. 2016;34(41):4905-12. doi: 10.1016/j.vaccine.2016.08.067. PubMed PMID: 27577556.

37. Pebody R, Warburton F, Ellis J, Andrews N, Potts A, Cottrell S, et al. Effectiveness of seasonal influenza vaccine for adults and children in preventing laboratory-confirmed influenza in primary care in the United Kingdom: 2015/16 end-of-season results. Euro Surveill. 2016;21(38). doi: 10.2807/1560-7917.ES.2016.21.38.30348. PubMed PMID: 27684603.

38. Skowronski DM, Chambers C, Sabaiduc S, De Serres G, Winter AL, Dickinson JA, et al. Beyond Antigenic Match: Possible Agent-Host and Immuno-epidemiological Influences on Influenza Vaccine Effectiveness During the 2015-2016 Season in Canada. J Infect Dis. 2017;216(12):1487-500. doi: 10.1093/infdis/jix526. PubMed PMID: 29029166; PubMed Central PMCID: PMCPMC5853508.

39. Jackson ML, Chung JR, Jackson LA, Phillips CH, Benoit J, Monto AS, et al. Influenza Vaccine Effectiveness in the United States during the 2015-2016 Season. N Engl J Med. 2017;377(6):534-43. doi: 10.1056/NEJMoa1700153. PubMed PMID: 28792867.

40. Pebody R, Warburton F, Ellis J, Andrews N, Potts A, Cottrell S, et al. End-of-season influenza vaccine effectiveness in adults and children, United Kingdom, 2016/17. Euro Surveill. 2017;22(44). doi: 10.2807/1560-7917.ES.2017.22.44.17-00306. PubMed PMID: 29113630; PubMed Central PMCID: PMCPMC5710133.

41. Flannery B, Chung JR, Monto AS, Martin ET, Belongia EA, McLean HQ, et al. Influenza Vaccine Effectiveness in the United States During the 2016-2017 Season. Clin Infect Dis. 2019;68(11):1798-806. doi: 10.1093/cid/ciy775. PubMed PMID: 30204854; PubMed Central PMCID: PMCPMC6522684.

42. Kissling E, Nunes B, Robertson C, Valenciano M, Reuss A, Larrauri A, et al. I-MOVE multicentre case-control study 2010/11 to 2014/15: Is there within-season waning of influenza type/subtype vaccine effectiveness with increasing time since vaccination? Euro Surveill. 2016;21(16). doi: 10.2807/1560-7917.ES.2016.21.16.30201. PubMed PMID: 27124420.
